# Evaluation of the Surface Properties of Three CAD/CAM Ceramics: A Comparative In Vitro Study

**DOI:** 10.3390/dj13120550

**Published:** 2025-11-22

**Authors:** Cristian Boanca, Kamel Earar, Sergiu Ciprian Focsaneanu, Dorin Ioan Cocoș, Cristian Constantin Budacu

**Affiliations:** 1 Faculty of Dental Medicine, “Dunărea de Jos” University, 800008 Galați, Romania; andiboanca@yahoo.com; 2 Faculty of Material Science and Engineering, University Politehnica of Bucharest, 313 Splaiul Independentei, District 6, 060042 Bucharest, Romania; focsaneanu.sergiu@gmail.com; 3 Faculty of Dental Medicine, “Grigore T. Popa” University of Medicine and Pharmacy from Iasi, 16 Universitatii Str., 700115 Iasi, Romania; cristibudacu@yahoo.com

**Keywords:** CAD/CAM, ceramic materials, dental restoration, energy-dispersive X-ray spectroscopy (EDS), scanning electron microscopy (SEM), surface properties, wettability

## Abstract

**Aim:** This study aimed to evaluate and compare the surface properties, roughness, skewness, and wettability of three CAD/CAM-processed ceramic materials: zirconia-reinforced lithium silicate (Celtra Duo), feldspathic ceramic (CEREC Blocs), and lithium disilicate (CEREC Tessera). **Materials and Methods:** Thirty specimens (10 per group) were fabricated (10 × 10 × 2 mm) using the CEREC Omnicam AC system with the MC XL milling unit and SpeedFire furnace. Surface characterization included computed tomography (CT) for dimensional accuracy, scanning electron microscopy (SEM) with energy-dispersive X-ray spectroscopy (EDS) for microstructure and composition, profilometry for roughness parameters (Ra, Rq, Rsk), and contact angle measurements with water, ethylene glycol, and diiodomethane for wettability. Statistical analysis was performed using ANOVA (*p* < 0.05). **Results:** No dimensional or compositional changes occurred after processing. Mean Ra values were 2.8647 µm for lithium disilicate, 1.9715 µm for zirconia-reinforced lithium silicate, and 1.9148 µm for feldspathic ceramic. All materials exhibited negative skewness, with zirconia-reinforced lithium silicate showing the smoothest morphology. Contact angle results indicated greater hydrophilicity for lithium disilicate (31.41°), followed by feldspathic ceramic (38.31°) and zirconia-reinforced lithium silicate (42.48°). **Conclusions:** Zirconia-reinforced lithium silicate demonstrated a favorable combination of surface topography, roughness, and wettability, suggesting superior long-term clinical performance.

## 1. Introduction

Restorative dentistry is a comprehensive approach that focuses on diagnosing, examining, and effectively treating affections of the teeth and their supporting structures. The goal of dental restorations is not only to regain functionality but also the aesthetic appeal, ultimately enhancing a patient’s overall oral health and quality of life [1,2]. According to global overviews and forecasts by MarketsandMarketsTM, the restorative dentistry market is expected to grow by 6.3% to nearly 27.37 billion USD from 2024 to 2030. In this context, there is a considerable need for the development of materials and processing techniques that yield dental prostheses with improved physico-chemical, biological, and aesthetic characteristics [3].

Despite being more affordable, traditional methods of fabricating dental restorations are time-consuming and may have low accuracy since they involve a large amount of manual crafting. Moreover, the patients require temporary restorations since the customized ones cannot be provided immediately [2]. As a solution to this issue, starting in the 1980s, computer-aided design (CAD)/computer-aided manufacturing (CAM) technology was introduced in dentistry. CAD/CAM allows the creation of well-defined two-dimensional and three-dimensional dental restorations via additive, subtractive, or hybrid techniques [4]. The CAD/CAM workflow involves three main steps: (1) intraoral scanning to capture the precise morphology of the oral cavity, (2) digital modeling of the restoration using specialized software, and (3) fabrication by subtractive milling, additive layering, or a hybrid approach [5,6]. Besides providing long-lasting, aesthetically pleasing, and highly personalized restorations, CAD/CAM technology is associated with a higher level of patient satisfaction in terms of “restoration production time” and “natural feeling” [3,7,8].

An important aspect to be considered when designing dental restorations is the material selection. Currently, the gold standard is represented by metal-ceramic crowns due to their longevity and appropriate physico-chemical and biological properties. Nevertheless, in time, the metallic substructure leads to greening of the gingivae and consequently inferior aesthetic features [9]. Studies showed that ceramic materials are the optimal choice for natural-looking restoration, and research is constantly being conducted to improve the mechanical features of these materials to combine aesthetics with resilience [10,11]. The most used ceramics in dentistry are zirconia, glass-ceramics, and resin-ceramics. Zirconia is highly biocompatible, but zirconia-only crowns present a high risk of breakage and are generally more expensive compared to other ceramics [12,13]. On the other hand, resin-ceramics, consisting of a polymeric matrix with inorganic refractory fillers, have lower color reliability, especially after polishing or treatment with sealing agents [14]. Therefore, glass-ceramics are considered the most balanced ones as they provide high thermal and mechanical resistance, exceptional aesthetics, and high biocompatibility [15].

Another significant aspect is related to the surface properties of the material, as they have a great influence on the bonding, mechanical strength, and wear resistance, all of which finally influence the clinical performance of the restorations [16,17]. Surface roughness, for example, is a key quality factor for the restoration, a smooth surface increasing the modulus of rupture, thus reducing the fracture risk and the abrasion with opposing teeth, and limiting bacterial adhesion [18]. Still, an optimal roughness must be preserved to ensure the integration of the material in the surrounding tissue [19,20].

For these reasons, the evaluation of the surface characteristics of dental ceramics is essential, the available literature data on this subject being quite limited. The purpose of this study was the comparative analysis of the surface properties of three consecrated ceramic materials-zirconia-reinforced lithium silicate, feldspathic ceramic, and lithium disilicate. Feldspathic porcelains, comprising quartz, feldspar, and kaolin, are considered pioneer ceramics and are amongst the most used materials in restorative dentistry. They present superior optical properties, good chemical stability, and biocompatibility, but a major disadvantage of feldspaths is their failure tendency due to chipping, fracture, debonding, or microleakage [21]. In response to these issues, their chemical composition was modified by the incorporation of lithium silicate crystals, which led to an improvement of mechanical properties without compromising the optical ones [22,23,24,25]. Later, lithium silicate alone was also introduced in the dental restoration field, initially being intended only for use with press technology. Nowadays, lithium silicate-based ceramic blocks for CAD/CAM are available on the market and are comprising 40% platelet-shaped lithium meta-silicate crystals embedded in a bluish-colored glassy matrix. To obtain a natural tooth-resembling color and an appropriate lithium silicate structure, a crystallization step is required before other processing techniques, the result being a material with good fracture resistance and mechanical strength [23]. For a further improvement of the mechanical properties, zirconia-reinforced lithium disilicate consisting of a lithium-metasilicate glass–ceramic matrix with approximately 8–12% zirconium dioxide grains was also developed [24,26,27].

Recent studies have emphasized the need for updated comparative analyses of CAD/CAM ceramics to better understand how differences in composition and microstructure influence surface behavior and clinical performance [3,4].

Given the limited comparative data on how CAD/CAM processing influences the surface behavior of clinically relevant ceramics, this study aimed to perform a comprehensive evaluation of zirconia-reinforced lithium silicate, feldspathic ceramic, and lithium disilicate materials. Although feldspathic ceramics exhibit excellent aesthetics and chemical stability, their mechanical limitations have led to the development of lithium silicate-based and zirconia-reinforced formulations, which offer enhanced fracture resistance without compromising translucency. However, there is still no consensus regarding how differences in composition and microstructure affect key surface characteristics critical for adhesion and clinical performance.

***Therefore, this* in vitro *study aimed to:***

1. Evaluate and compare the surface morphology of zirconia-reinforced lithium silicate, feldspathic ceramic, and lithium disilicate CAD/CAM materials using scanning electron microscopy (SEM).
2. Analyze the surface roughness parameters (Ra, Rq, Rsk) obtained through 3D profilometry.
3. Assess surface wettability and free surface energy using contact angle measurements.
4. Determine the elemental composition of each material through energy-dispersive X-ray spectroscopy (EDS).

The null hypothesis of this study was that no significant differences would be observed in the surface roughness, wettability, or elemental composition among the three tested CAD/CAM ceramics after milling.

## 2. Materials and Methods

### 2.1. Study Design and Setting

This was an experimental in vitro comparative study conducted between March and July 2024 in the Research Laboratory of the Faculty of Dental Medicine, “Grigore T. Popa” University of Medicine and Pharmacy, Iași, Romania, in collaboration with the Faculty of Material Science and Engineering, University Politehnica of Bucharest.

### 2.2. CAD/CAM Processing of the Ceramic Materials

Three types of CAD/CAM ceramic blocks were used: zirconia-reinforced lithium silicate (Celtra Duo, Dentsply Sirona, Bensheim, Germany), feldspathic ceramic (CEREC Blocs, Dentsply Sirona, Bensheim, Germany), and lithium disilicate (CEREC Tessera, Dentsply Sirona, Bensheim, Germany). Each material was processed by CAD/CAM technology to fabricate fixed prosthetic restorations. The restorations were obtained using a CEREC Sirona Omnicam AC system (Dentsply Sirona, Bensheim, Germany), equipped with an MC XL milling unit, Radium module, and SpeedFire sintering furnace.

**Figure 1:**
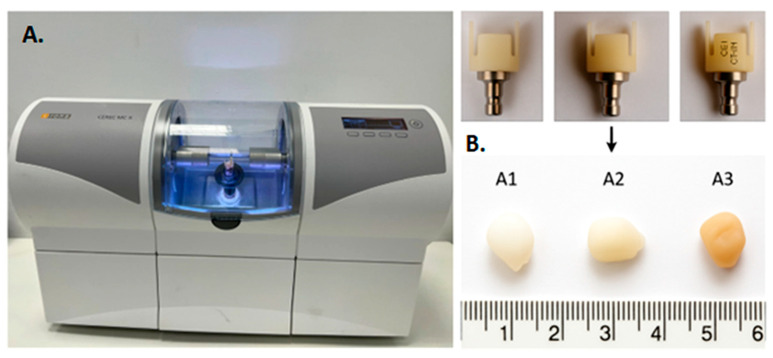
(**A**,**B**) CAD/CAM milling procedure and resulting ceramic restorations: (**A**): CEREC MC XL milling unit; (**B**): Macroscopic images of the ceramic blocks after CAD/CAM milling and the resulting restorations (**A1**–**A3**). Macroscopic images of the three ceramic blocks after CAD/CAM milling and the corresponding restorations: (**A1**) zirconia-reinforced lithium silicate (Celtra Duo), (**A2**) feldspathic ceramic (CEREC Blocs), and (**A3**) lithium disilicate (CEREC Tessera). The samples illustrate the dimensional precision and surface quality achieved through CAD/CAM processing, highlighting the absence of visible defects or irregularities.

Image acquisition, digital modeling, and milling were performed using CEREC Software (version 5.2.3, Dentsply Sirona, Bensheim, Germany). The resulting specimens were labeled as A1 (Celtra Duo), A2 (CEREC Blocs), and A3 (CEREC Tessera). After fabrication, all specimens were numerically coded and randomly assigned to the different analyses (CT, SEM/EDS, profilometry, and contact angle measurements) using a simple randomization procedure to minimize operator bias and ensure balanced sample allocation. The macroscopic aspect of the fabricated restorations is presented in Figure 1.

The main characteristics of the tested CAD/CAM ceramic materials are presented in Table 1.

### 2.3. Computed Tomography (CT) Analysis

Dimensional evaluation and detection of possible defects, such as porosities or inclusions, were performed using a Nikon XT H 225 micro-CT system (Nikon Metrology, Irvine, CA, USA). Three-dimensional reconstructions were generated with CT 3D software (Nikon Metrology, Irvine, CA, USA), and image analysis was carried out using VGStudio Max (Volume Graphics GmbH, Heidelberg, Germany). Each ceramic group (A1, A2, A3) included three samples examined under identical scanning parameters. The specimen size (*n* = 3 per material) was selected based on previous studies evaluating surface properties of CAD/CAM ceramics with a similar experimental design and analytical methodology [7,16].

### 2.4. Scanning Electron Microscopy (SEM) and Energy-Dispersive X-Ray Spectroscopy (EDS)

Surface morphology and elemental composition were analyzed using a Quanta Inspect F scanning electron microscope (FEI Company, Eindhoven, The Netherlands), operating at 30 kV acceleration voltage and 10 mm working distance. The system was equipped with an EDS detector (EDAX Inc., Mahwah, NJ, USA; 132 eV resolution at MnKα) for micro-elemental analysis. For each ceramic group, three specimens were examined in randomly selected surface areas. Each EDS spectrum was acquired over an area of approximately 50 × 50 μm, ensuring representative sampling of the surface microstructure.

### 2.5. Surface Roughness Analysis

Surface roughness was measured with a Form Talysurf I-Series PRO profilometer (Taylor Hobson Ametek, Aurora, IL, USA) fitted with a standard probe transducer. Five readings were taken from different regions of each sample, and the data were processed using Metrology 4.0 software (Taylor Hobson Ametek, Aurora, IL, USA). The following parameters were recorded:

- Ra—arithmetic mean roughness;
- Rq—root mean square roughness;
- Rt—total height of the roughness profile.

### 2.6. Wettability Measurements

Contact angles were determined using a Krüss Drop Shape Analyzer DSA100 (A. Krüss Optronic GmbH, Hamburg, Germany). Three test liquids were used: distilled water (W), ethylene glycol (EG), and diiodomethane (DIM). For each liquid, five consecutive measurements were performed on both the internal and external surfaces of the samples.

The contact angle images were processed with AxioVision software (SE64 Rel. 4.9.1, Carl Zeiss Microscopy GmbH, Heidelberg, Germany), and the free surface energy (FSE) was calculated according to Young’s equation. All measurements were performed under controlled laboratory conditions at 23 ± 1 °C and 50 ± 5% relative humidity. Surface free energy (γs) and its dispersive (γs^d) and polar (γs^p) components were calculated using the Owens–Wendt method with water and diiodomethane as probe liquids (γl/γl^d/γl^p in mN/m: water 72.8/21.8/51.0; diiodomethane 50.8/50.8/0).

### 2.7. Investigators

All CAD/CAM specimen fabrication procedures were performed by an experienced dental technician under the supervision of the research team. All experimental testing (CT, SEM/EDS, profilometry, and contact angle analysis) was conducted independently by two trained investigators, and measurements were repeated to ensure data reproducibility. During data acquisition and analysis, the investigators were blinded to the material type to minimize bias in image interpretation and measurement processing. Operator bias was controlled by performing all measurements independently by two calibrated investigators, with equipment recalibrated before each testing session, and data processing carried out using standardized software algorithms.

### 2.8. Statistical Analysis

Data were expressed as mean ± standard deviation (SD). Statistical analysis was performed using GraphPad Prism 10.0 software (GraphPad Software Inc., San Diego, CA, USA). Normality was assessed using the Shapiro–Wilk test. Differences among groups were evaluated with one-way ANOVA, followed by Tukey’s post hoc test, both performed in the same software environment. A *p*-value < 0.05 was considered statistically significant.

## 3. Results

### 3.1. EDS

**Figure 2:**
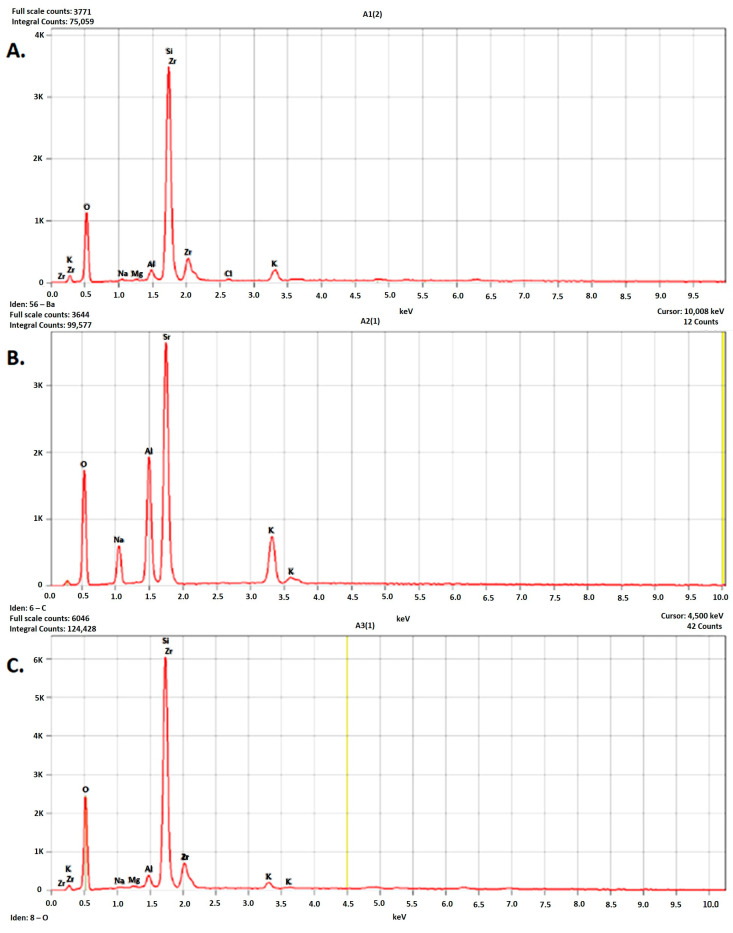
EDS spectra of the analyzed materials—Celtra Duo (**A**), CEREC Blocs (**B**), and CEREC Tessera (**C**). Energy-dispersive X-ray spectroscopy (EDS) elemental composition of the zirconia-reinforced lithium silicate ceramic (A1), reported in both weight percent (wt%) and atomic percent (at%). The primary constituents were oxygen (53.84 wt%) and silicon (30.54 wt%), confirming a glass-ceramic matrix structure with a high silicate content. Notably, zirconium was present at 10.33 wt%, representing the reinforcing crystalline phase characteristic of this material class. Minor levels of aluminum (1.59 wt%) and potassium (2.42 wt%) were also detected, alongside trace amounts of sodium, magnesium, and chlorine, which are typically associated with flux additives and glass modifiers introduced during ceramic processing. The presence of zirconium in measurable quantities distinguishes this composition from non-reinforced feldspathic ceramics and correlates with the improved mechanical performance reported for zirconia-reinforced lithium silicate formulations.

The EDS spectra of all analyzed materials showed that the main elements were oxygen and silicon (Figure 2).

In the feldspathic ceramic (A2), aluminum, sodium, and potassium were identified in notable amounts (Table 2).

The two lithium silicate-based ceramics (A1 and A3) showed similar elemental compositions (Table 3 and Table 4).

The presence of zirconium in A3 was attributed to an equipment artifact, as zirconium is not part of the CEREC Tessera block.

The EDS analysis indicated that the elemental composition remained unchanged after CAD/CAM processing, confirming that this technique did not alter the chemical integrity of the materials.

### 3.2. CT

Computed tomography revealed no evidence of porosity or other structural defects that could affect the functionality of the restorations. All samples exhibited accurate anatomical morphology and dimensional conformity with the digital design.

The dimensional analysis showed similar values among the three crowns, demonstrating high precision of the CAD/CAM milling process.

### 3.3. SEM

**Figure 3:**
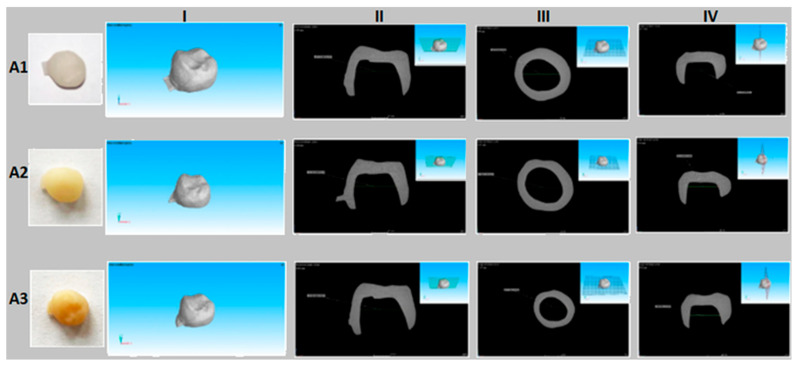
CT images of the analyzed samples. CT images of CAD/CAM-processed ceramic restorations: (**A1**,**I**–**IV**) zirconia-reinforced lithium silicate, (**A2**,**I**–**IV**) feldspathic ceramic, and (**A3**,**I**–**IV**) lithium disilicate.

The SEM images of the three materials at different magnifications (100×, 500×, and 10,000×) are presented in Figure 3. All samples showed a characteristic milled surface with moderate roughness and no visible porosities or microfractures.

At 500× magnification, lamellar structures were observed in both zirconia-reinforced lithium silicate (A1) and lithium disilicate (A3), randomly oriented within a glassy matrix.

At 10,000× magnification, zirconia crystallite clusters embedded in the glassy matrix were evident in A1, contributing to its higher surface irregularity.

Feldspathic ceramic (A2) displayed a more homogeneous surface morphology with irregular, interconnected structures dispersed in a vitreous matrix.

**Figure 4:**
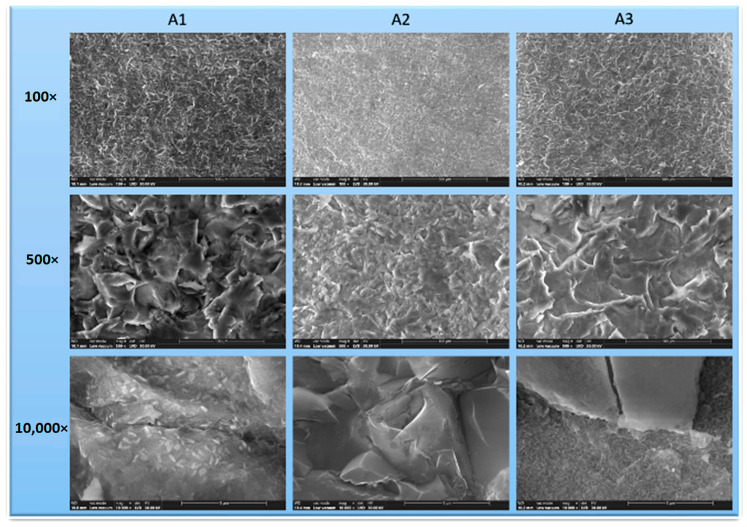
SEM images of the analyzed samples at different magnification scales. SEM micrographs of CAD/CAM-processed ceramics (**A1**–**A3**) at different magnifications. The images reveal typical milling patterns, lamellar structures within glassy matrices, and moderate surface roughness, without visible porosities or microfractures, confirming adequate surface morphology.

Based on surface microstructure, the samples followed the roughness order A3 > A1 > A2, consistent with the profilometry results (Figure 4).

### 3.4. Surface Roughness

**Figure 5:**
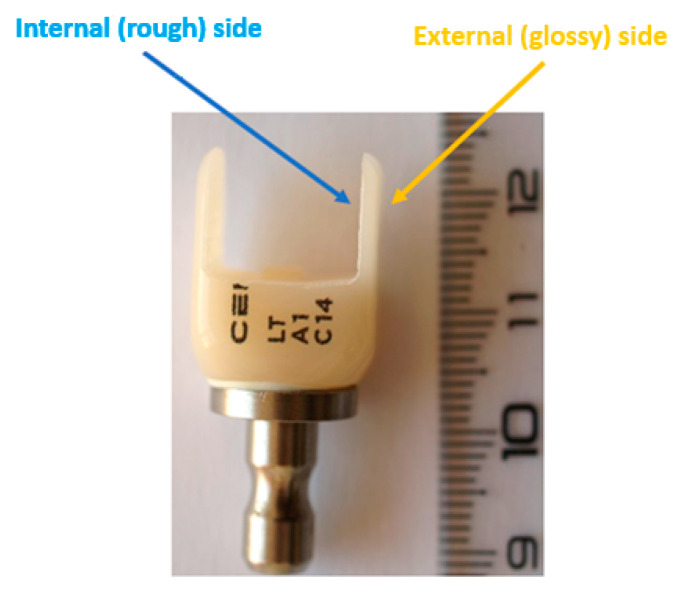
The internal (**rough**) side and the external (**glossy**) side of the remaining block material after CAD/CAM milling. Internal (milled) and external (non-milled) surfaces of the ceramic blocks after CAD/CAM processing. The internal surfaces mimic the adhesive interface of restorations, while the external sides illustrate the natural polished finish of unprocessed ceramic, highlighting differences in topography and roughness.

**Figure 6:**
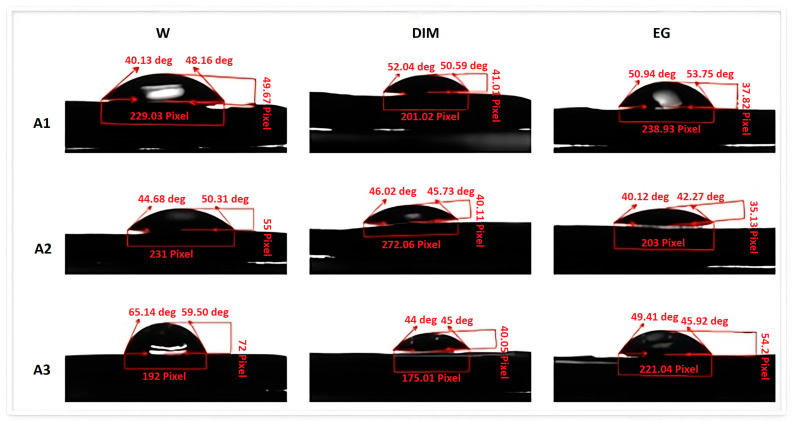
Contact Angle Measurements of CAD/CAM-Processed Dental Ceramics Using Three Probe Liquids. Sessile drop images showing contact angles with water, ethylene glycol, and diiodomethane on ceramic surfaces (A1–A3). These measurements were used to calculate surface free energy parameters according to the Owens–Wendt method. A1, A2, and A3 correspond to the three CAD/CAM ceramic materials analyzed in the study: A1—zirconia-reinforced lithium silicate, A2—feldspathic ceramic, and A3—lithium disilicate.

To comparatively analyze the surfaces of CAD/CAM restorations and those of the remaining block material, measurements were performed on the samples (A1–A3) and on both the internal (milled) and external (non-milled) sides (Figure 5 and Figure 6).

**Figure 7:**
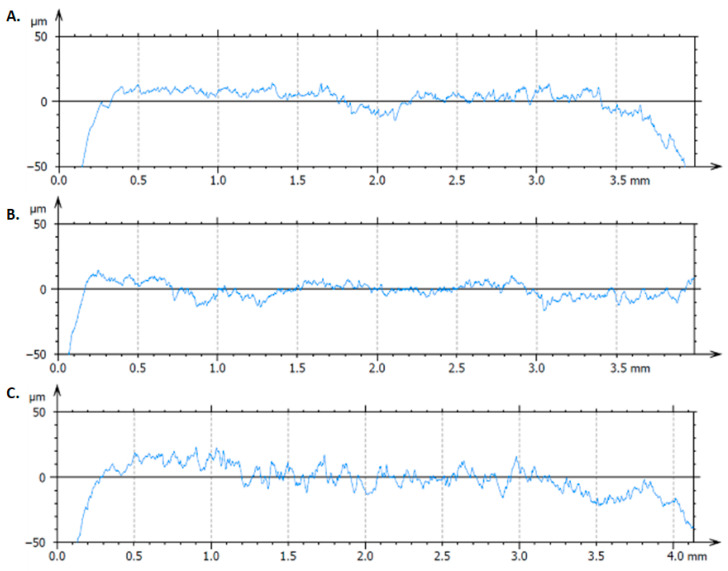
Representative roughness profiles of the analyzed samples. Representative roughness profiles of CAD/CAM-processed ceramic restorations: (**A**) zirconia-reinforced lithium silicate, (**B**) feldspathic ceramic, and (**C**) lithium disilicate. The graphs illustrate the surface topography obtained by profilometric analysis.

The profilometry results (Figure 7, Table 5) showed that the mean Ra values followed the descending order A3 > A1 > A2 (2.8647, 1.9715, and 1.9148 μm, respectively).

For Rq, the order was A3 > A2 > A1 (4.3593, 2.8214, and 2.7405 μm, respectively).

All materials exhibited negative Rsk values, with the least negative value recorded for A1 (−1.1601), followed by A2 (−2.2669) and A3 (−2.8007).

In all cases, the restoration surface (sample) showed the highest roughness, followed by the internal side of the ceramic block, while the lowest values were recorded on the external side.

These differences reflected the surface topography produced by the CAD/CAM milling process, the complex geometry of the crowns contributing to the increased surface irregularity.

### 3.5. Contact Angle

The contact angle and surface free energy (γs) analyses, calculated using the Owens–Wendt approach, are presented in Table 6. All tested materials exhibited moderate wettability for the three test liquids, with higher hydrophilicity on the internal (milled) surfaces compared to the external (non-milled) ones (Figure 5).

The contact angle values followed the order A3 < A2 < A1 (31.41°, 38.31°, and 42.48°, respectively), corresponding to increasing hydrophobicity from lithium disilicate to zirconia-reinforced lithium silicate.

FSE values displayed the reverse order A1 < A2 < A3, with the highest surface energy observed for A3, indicating greater surface polarity and higher potential for adhesive bonding.

Table 6 reports the static contact angle values (°) expressed as mean ± standard deviation (SD) and the corresponding surface free energy parameters (mN/m) calculated using the Owens–Wendt method. The total surface free energy (γs) is the sum of its dispersive (γs^d) and polar (γs^p) components. Lower contact angles correlated with higher γs values, indicating greater surface polarity and improved wettability, particularly for lithium disilicate (A3).

### 3.6. Overall Inference

Overall, among the tested CAD/CAM ceramics, zirconia-reinforced lithium silicate (A1) showed the most balanced surface behavior, combining moderate roughness, stable chemical composition, and optimal wettability for adhesive bonding. Lithium disilicate (A3) presented the highest surface irregularity and hydrophilicity, while feldspathic ceramic (A2) exhibited the smoothest and least hydrophilic surface. These findings confirm clear structural and surface-related differences among the three materials.

These results can guide clinicians in selecting appropriate CAD/CAM ceramics based on surface properties to enhance the performance and longevity of dental restorations.

## 4. Discussion

### 4.1. General Findings

The findings of this study highlight the importance of understanding the surface properties of ceramic materials processed through CAD/CAM technology. All three tested ceramics, zirconia-reinforced lithium silicate, feldspathic ceramic, and lithium disilicate, maintained their chemical composition and dimensional stability after milling. These results confirm the well-known advantages of CAD/CAM systems, which ensure high reproducibility, precision, and predictable outcomes in restorative dentistry [2,5,6]. From a clinical standpoint, the digital workflow reduces the variability associated with manual laboratory techniques, minimizing errors and improving the reliability of ceramic restorations [3,7]. Similar observations were reported by Güth et al. [4], who demonstrated that CAD/CAM fabrication preserves the microstructural integrity of ceramics while maintaining excellent fit accuracy.

### 4.2. Surface Morphology and Roughness

Surface roughness is a key determinant of the clinical performance of prosthetic restorations, influencing wear resistance, bacterial adhesion, and aesthetic stability [16,18]. In the present study, profilometry and SEM analyses revealed moderate roughness across all samples, with lithium disilicate showing the highest Ra and Rq values. These results are consistent with previous reports [21,23,26,27], which collectively demonstrated that lithium disilicate ceramics tend to develop more pronounced surface irregularities after CAD/CAM milling due to their crystalline microstructure, whereas feldspathic ceramics exhibit smoother surfaces because of their finer glass matrix. Despite this higher roughness, lithium disilicate remains widely used for its superior fracture toughness, optical stability, and long-term clinical reliability. The negative skewness (Rsk) values observed for all samples indicate that the surfaces were dominated by valleys rather than peaks. Clinically, this surface morphology favors resin cement penetration, enhances micromechanical interlocking, and minimizes stress concentration at the adhesive interface, thereby improving the long-term stability of the bond. The negative skewness values obtained for all samples also align with literature reports indicating that negatively skewed ceramic surfaces reduce friction and wear on opposing enamel [28]. Among the tested materials, zirconia-reinforced lithium silicate exhibited the most favorable skewness, corroborating data from Spitznagel et al. [22] who demonstrated improved wear behavior and surface homogeneity in zirconia-modified glass-ceramics.

### 4.3. Wettability and Surface Free Energy

Wettability, another crucial parameter, is closely related to bacterial colonization and bonding with resin-based cements [29,30]. In this study, the internal milled surfaces of the ceramic blocks displayed higher hydrophilicity compared to the external polished surfaces, which agrees with the findings of Alrahlah et al. [19] and Zhang et al. [20] who observed that machining processes increase surface energy and enhance adhesion. However, excessive hydrophilicity can also favor bacterial accumulation [29]. Zirconia-reinforced lithium silicate exhibited the lowest wettability among the tested materials, a result consistent with that reported by Campos et al. [24], who found that zirconia incorporation reduces surface energy but improves chemical stability.

### 4.4. Elemental Composition

The EDS analysis confirmed that CAD/CAM processing did not modify the elemental composition of the materials, which agrees with the findings of Denry and Kelly [16,17] showing that subtractive digital fabrication preserves chemical integrity. Therefore, any variations in performance among the studied ceramics are attributed to their intrinsic structural and microcrystalline features rather than compositional alterations induced by processing.

### 4.5. Comparative Assessment and Clinical Significance

When comparing the three ceramic systems, feldspathic ceramics exhibited the smoothest surface and balanced wettability, like the results of Egilmez et al. [21] who emphasized their superior optical behavior. However, their mechanical fragility limits their use in high-stress regions [22]. Conversely, lithium disilicate demonstrated greater surface roughness but superior mechanical properties and clinical longevity [9,23] while zirconia-reinforced lithium silicate provided an intermediate balance between the two, confirming findings reported by Elsaka et al. [24] regarding its optimized strength and esthetic compromise.

Clinically, these variations in surface roughness and wettability influence plaque retention, discoloration, and adhesive performance [27,29]. The present findings are comparable with those of Stawarczyk et al. [14], who demonstrated that surface finishing significantly affects adhesion and longevity of ceramic restorations. Our results also support the recommendation that adhesive strategies should be material-specific: lithium disilicate benefits from etching and silanization, whereas zirconia-based ceramics require air-abrasion or specialized primers to ensure durable bonding [12,24].

### 4.6. Clinical Implications

The results of this study offer practical guidance for material selection in restorative dentistry. Feldspathic ceramics, owing to their superior translucency, are recommended for anterior veneers and minimally invasive restorations where aesthetics is the main concern. Lithium disilicate, with its higher strength and toughness, is preferable for single crowns and short-span fixed partial dentures. Zirconia-reinforced lithium silicate provides a balanced combination of strength and optical quality, making it suitable for both anterior and posterior restorations produced through CAD/CAM workflows.

### 4.7. Strengths, Limitations, and Future Perspectives

Overall, the present study adds to existing literature confirming that CAD/CAM processing maintains both chemical stability and clinically acceptable surface characteristics of modern dental ceramics [2,5,6]. Each material exhibits distinct advantages: feldspathic ceramics provide superior translucency, lithium disilicate offers enhanced mechanical durability, and zirconia-reinforced lithium silicate ensures an optimal balance between strength and esthetics [9,23,24]. These findings collectively support the selection of CAD/CAM ceramics based on specific clinical indications and underscore the importance of appropriate surface conditioning to maximize restoration longevity. Future studies should further investigate the correlation between surface morphology, adhesive behavior, and long-term in vivo performance.

The present findings have clear clinical translational value, as they provide evidence supporting the selection of CAD/CAM ceramics based on their surface properties, which directly influence adhesion, bacterial colonization, and restoration longevity. The study demonstrates that zirconia-reinforced lithium silicate and lithium disilicate ceramics offer favorable surface characteristics for durable, aesthetic restorations when properly conditioned before cementation.

The main methodological strengths include the use of advanced analytical techniques, SEM, EDS, profilometry, and contact angle measurements, which ensured accurate and reproducible evaluation of microstructural and surface parameters. The combination of quantitative and qualitative analyses strengthens the validity of comparative results among the three CAD/CAM ceramic systems.

However, the study has certain limitations, primarily related to its in vitro design and the absence of mechanical and fatigue testing, which may influence real-world clinical performance. Additionally, the results were obtained under controlled laboratory conditions, which may not fully replicate the complex oral environment.

Despite its strengths, several limitations must be acknowledged in more detail. First, the in vitro setting cannot simulate the thermal, mechanical, and chemical variations in the oral cavity, which could affect the long-term stability of ceramics. Second, the relatively small sample size and the use of a single CAD/CAM system may limit the generalizability of the results to other manufacturing setups. Third, no evaluation of adhesive bond strength or fatigue resistance was performed, parameters that are critical for predicting clinical performance. Finally, the study did not explore the influence of different surface treatments or cementation procedures, which are known to significantly affect ceramic behavior. These aspects should be addressed in future investigations to validate and expand the current findings.

Future research should include long-term in vivo studies correlating surface characteristics with clinical outcomes such as bond strength, bacterial accumulation, wear resistance, and color stability. Expanding this investigation to include different surface treatments and cementation protocols could further enhance the clinical applicability of CAD/CAM ceramics. In addition, future in vivo comparative studies and cyclic loading tests are recommended to evaluate the long-term mechanical durability and surface stability of these materials under simulated oral conditions.

## 5. Conclusions

Within the limitations of this in vitro study, it can be concluded that all three CAD/CAM ceramics exhibited moderate surface roughness with negative skewness profiles, features that favor adhesive bonding. Lithium disilicate presented the highest Ra and Rq values, whereas feldspathic ceramics showed the smoothest surfaces. Regarding wettability, internal milled areas displayed higher hydrophilicity than external polished ones, and zirconia-reinforced lithium silicate demonstrated the lowest wettability, consistent with its superior chemical stability. EDS analysis confirmed that CAD/CAM processing did not alter the elemental composition of the tested materials, reflecting their excellent thermal and chemical stability. Clinically, feldspathic ceramics offer the best translucency, lithium disilicate provides superior mechanical strength, and zirconia-reinforced lithium silicate achieves an optimal balance between esthetics and durability.

## Figures and Tables

**Table 1:** Characteristics of the tested CAD/CAM ceramic materials and processing parameters.

Code	Brand Name	Manufacturer	Composition/Type	LOT No.	Milling Machine	Software	Thermal/Sintering Process
**A1**	Celtra Duo	Dentsply Sirona, Bensheim, Germany	Zirconia-reinforced lithium silicate glass-ceramic (ZLS)	-	CEREC MC XL (Dentsply Sirona, Bensheim, Germany)	CEREC Software (version 5.2.3, Dentsply Sirona)	Crystallized in the SpeedFire furnace at 840 °C for 10 min
**A2**	CEREC Blocs	Dentsply Sirona, Bensheim, Germany	Feldspathic glass-ceramic (SiO_2_-Al_2_O_3_-K_2_O-Na_2_O)	-	CEREC MC XL (Dentsply Sirona, Bensheim, Germany)	CEREC Software (version 5.2.3, Dentsply Sirona)	No additional firing required
**A3**	CEREC Tessera	Dentsply Sirona, Bensheim, Germany	Lithium disilicate glass-ceramic reinforced with virgilite crystals	-	CEREC MC XL (Dentsply Sirona, Bensheim, Germany)	CEREC Software (version 5.2.3, Dentsply Sirona)	Fired in the SpeedFire furnace at 760 °C for 4 min

**Table 2:** EDS elemental composition of the A1 sample (zirconia-reinforced lithium silicate). The table displays the quantitative elemental distribution in zirconia-reinforced lithium silicate (A1) as determined by EDS. Silicon and oxygen predominate, reflecting the glass-ceramic matrix, while zirconium (10.33 wt%) represents the reinforcing crystalline phase that contributes to increased strength and fracture resistance. Minor levels of potassium, aluminum, and trace elements indicate typical fluxing additives introduced during ceramic processing.

Element	Weight %	Weight % Error	Atom %	Atom % Error
**O**	53.84	±0.62	71.04	±0.81
**Na**	0.75	±0.07	0.69	±0.07
**Mg**	0.22	±0.06	0.19	±0.05
**Al**	1.59	±0.10	1.24	±0.08
**Si**	30.54	±0.22	22.96	±0.17
**Cl**	0.32	±0.04	0.19	±0.03
**K**	2.42	±0.06	1.31	±0.03
**Zr**	10.33	±1.30	2.39	±0.30
**Total**	100.00		100.00	

**Table 3:** EDS elemental composition of the A2 sample. Energy-dispersive X-ray spectroscopy (EDS) results of the feldspathic ceramic sample (A2), showing the elemental composition expressed in both weight percent (wt%) and atomic percent (at%). The material is primarily composed of oxygen (48.11 wt%) and silicon (26.70 wt%), consistent with its classification as a silica-based glass ceramic. Aluminum (12.40 wt%) and sodium (7.30 wt%) were also identified in considerable proportions, reflecting the presence of aluminosilicate glass phases. Potassium (5.49 wt%) was detected in a lower concentration, likely associated with its role as a fluxing oxide during ceramic processing. The compositional distribution correlates with the typical mineralogical constituents of feldspathic ceramics, which are characterized by a high silica-glass matrix enriched with alkali and alkaline-earth modifiers. The absence of zirconium or other radiopaque elements is in line with the non-reinforced nature of this sample (A2), distinguishing it from the lithium silicate-based materials (A1, A3) presented elsewhere in the study.

Element	Weight %	Weight % Error	Atom %	Atom % Error
**O**	48.11	±0.45	61.68	±0.58
**Na**	7.30	±0.12	6.51	±0.11
**Al**	12.40	±0.14	9.43	±0.10
**Si**	26.70	±0.20	19.50	±0.14
**K**	5.49	±0.10	2.88	±0.05
**Total**	100.00		100.00	

**Table 4:** EDS elemental composition of the A3 sample. EDS elemental composition of the lithium disilicate sample (A3). Oxygen (58.52 wt%) and silicon (30.91 wt%) were the predominant elements. Minor contents of aluminum (1.73 wt%), potassium (1.22 wt%), and zirconium (6.66 wt%) were also detected.

Element	Weight %	Weight % Error	Atom %	Atom % Error
**O**	58.52	±0.49	73.64	±0.61
**Na**	0.58	±0.10	0.51	±0.09
**Mg**	0.36	±0.05	0.30	±0.04
**Al**	1.73	±0.08	1.29	±0.06
**Si**	30.91	±0.19	22.16	±0.13
**K**	1.22	±0.04	0.63	±0.02
**Zr**	6.66	±0.80	1.47	±0.18
**Total**	100.00		100.00	

**Table 5:** Profilometry roughness parameters of the analyzed samples and internal and external sides of the remaining block material. Profilometric roughness parameters (Ra, Rq, Rsk) obtained for CAD/CAM-processed ceramic samples and their respective internal and external block surfaces. For each material (A1–A3), the internal and external surface roughness values are expressed relative to the machined sample (baseline), with percentage variations (Δ%) calculated for Ra and Rq. Among the tested ceramics, the lithium disilicate sample (A3) exhibited the highest mean surface roughness values (Ra = 2.86 µm, Rq = 4.36 µm), followed by zirconia-reinforced lithium silicate (A1) and feldspathic ceramic (A2), which showed comparatively lower Ra and Rq values. Notably, the external block surfaces across all materials demonstrated substantial reductions in roughness relative to the machined samples (up to −89.79% for Ra and −70.53% for Rq in A1), suggesting a smoother, less processed morphology. All samples and surfaces exhibited negative skewness (Rsk) values, indicating a predominance of valleys over peaks in the surface topography.

Set	Zona	Ra [µm]	Rq [µm]	Rsk	ΔRa % vs. Sample	ΔRq % vs. Sample	*p*-Value
A1	Sample	1.9715	2.7405	−1.1601			
A1	Internal side	1.8038	2.2854	−0.1891	−8.51%	−16.61%	ns
A1	External side	0.2013	0.8079	−11.9274	−89.79%	−70.52%	*p* < 0.05
A2	Sample	1.9148	2.8214	−2.2669			
A2	Internal side	1.7077	2.1848	−0.2938	−10.82%	−22.56%	ns
A2	External side	0.4326	0.6799	−1.8274	−77.41%	−75.90%	*p* < 0.05
A3	Sample	2.8647	4.3593	−2.8007			
A3	Internal side	1.8304	2.3576	−0.0663	−36.11%	−45.92%	ns
A3	External side	0.0766	0.1940	−10.4699	−97.33%	−95.55%	*p* < 0.05

“ns” refers to “not significant”.

**Table 6:** Static Contact Angle and Surface Free Energy (Owens–Wendt) Parameters of CAD/CAM Ceramic Surfaces.

Sample	Side	W (°) Mean ± SD	DIM (°) Mean ± SD	EG (°) Mean ± SD	γs^d (mN/m)	γs^p (mN/m)	γs (mN/m)
A1	External side	43.29 ± 7.08	50.23 ± 8.03	50.23 ± 5.44	34.1	24.9	59.0
A2	External side	46.30 ± 4.82	45.14 ± 4.84	42.31 ± 7.17	36.9	21.6	58.5
A3	External side	64.74 ± 4.19	45.35 ± 2.59	45.65 ± 4.91	36.8	10.9	47.7
A1	Internal side	42.49 ± 4.97	35.68 ± 5.74	39.10 ± 10.52	41.7	21.5	63.2
A2	Internal side	38.31 ± 4.37	37.06 ± 3.46	23.75 ± 7.98	41.1	24.1	65.1
A3	Internal side	31.41 ± 4.48	37.49 ± 3.21	23.25 ± 3.22	40.8	27.8	68.6

W, water; DIM, diiodomethane; EG, ethylene glycol; SD, standard deviation; γs^d, dispersive component of surface free energy; γs^p, polar component of surface free energy; γs, total surface free energy.

## Data Availability

The original contributions presented in this study are included in the article. Further inquiries can be directed to the corresponding authors.
